# Using Mendelian Randomisation to search for modifiable risk factors influencing the development of clonal haematopoiesis

**DOI:** 10.1038/s41408-024-01101-y

**Published:** 2024-07-16

**Authors:** Jessica M. Hislop, Molly Went, Charlie Mills, Amit Sud, Philip J. Law, Richard S. Houlston

**Affiliations:** 1 Division of Genetics and Epidemiology, The Institute of Cancer Research, London, UK; 2 Department of Medical Oncology, Dana-Farber Cancer Institute, Boston, MA USA; 3 Broad Institute of MIT and Harvard, Cambridge, MA USA; 4 Harvard Medical School, Boston, MA USA; 5 Centre for Immuno-Oncology, Nuffield Department of Medicine, University of Oxford, Oxford, UK

**Keywords:** Cancer genetics, Cancer genomics, Haematological cancer

Clonal haematopoiesis, the clonal expansion of a blood stem cell and its progeny, is increasingly detectable with age in the blood of healthy individuals [1, 2]. Clonal haematopoiesis of indeterminate potential (CHIP) is characterised by the expansion of clones with oncogenic mutations, primarily in *DNMT3A*, *TET2*, *ASXL1*, *JAK2*, and is associated with mosaic chromosomal alterations [2]. As well as being strongly linked to an increased risk of haematological cancers, CHIP has been associated with an increased risk of several non-haematological conditions, notably cardiovascular disease [2]. While CHIP is defined by its somatic mutation profile, which is influenced by heritable factors [1, 3], few studies have provided evidence for modifiable factors influencing CHIP and our understanding of its aetiological basis is so far limited. Observational studies have linked smoking to mosaic loss-of-Y chromosome (mLOY) and mutations in *ASXL1* [4, 5]. While tobacco smoke is a strong mutagen, observational associations can be confounded or the result of reverse causality [6, 7]. Moreover, the broader relationship between smoking and variants indicative of CHIP, and the extent to which residual confounding from correlated lifestyle factors may explain the observed relationship, is debated [4, 8].

Mendelian randomisation (MR) is an analytical strategy that uses germline genetic variants as instrumental variables (IVs) to infer potentially causal relationships [9]. Because genetic variants are randomly allocated at conception, the variants can be used as IVs for MR, which under certain assumptions (shown in Fig. 1) enables a natural experiment mimicking a randomised controlled trial. Compared with traditional observational study designs, MR studies are less susceptible to confounding and reverse causality, enabling estimation of putative potentially causal associations between exposures and outcomes [9].

**Fig. 1:**
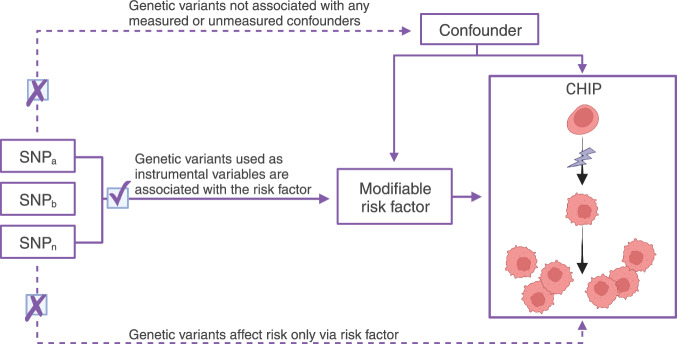
Principles of Mendelian Randomisation and the assumptions that need to be satisfied to derive unbiased causal effect estimates. Mendelian randomisation (MR) is used under three primary assumptions, specifically genetic variants are not associated with any confounder, genetic variants are associated with modifiable risk factors, and genetic variants influence the risk of developing CHIP through only the modifiable risk factor. The illustration shows a haematopoietic stem cell (HSC) undergoing a somatic mutation leading to increased HSC renewal and clonal haematopoiesis of indeterminate potential (CHIP). Created with BioRender.com.

We investigated potentially causal and modifiable risk factors for CHIP using a two-sample MR (2S-MR) framework, in which genetic variants associated with potential risk factors were first identified from genome-wide association studies (GWAS). We then assessed the association between the instrumental variables and CHIP in a large GWAS. As well as focusing on potentially modifiable dietary, lifestyle, obesity-related, inflammatory, and reproductive factors that have been implicated as risk factors for many cancers, we searched PubMed to identify additional factors that have been reported to influence the risk of haematological malignancies (Supplementary Table 1). Ethical approval was not required for this project as all the data came from summary statistics of published GWAS, and no individual-level data were used.

We performed 2S-MR using the TwoSampleMR package (https://github.com/MRCIEU/TwoSampleMR). We considered 7905 independent (*r*^2^ < 0.001, distance >10 Mb) genetic variants associated (*P* < 5 × 10^–8^) with 35 phenotypes, catalogued by MR-base (median = 17.0 IVs per trait, range = 1–524). For each genetic variant associated with potential risk factors, we extracted the corresponding effects for each outcome from a GWAS of autosomal mosaic chromosomal alterations (mCAaut), mosaic loss-of-X chromosome (mLOX), mLOY, and CHIP based on a meta-analysis of 454,803 UK BioBank (UKB) participants [3]. We report the numbers of cases and controls in Supplementary Table 4. We calculated *F*-statistics for each exposure-outcome pair and, to avoid weak-instrument bias [10], we included only those traits with a *F*-statistic >10. Furthermore, we only considered exposure traits if the proportion of variance explained (PVE) by the associated SNPs was greater than 0.1%. Estimates of the PVE were either obtained directly from publications or computed from the association statistics [11]. The median PVE explained by SNPs used as IVs for each of the 35 phenotypes examined as potential risk factors for CHIP was 4.9% (95% confidence interval (CI): 2.6–5.3%). The power of our study to demonstrate a causal association for CHIP is tabulated for each exposure in Supplementary Table 2. We only considered continuous traits, as analysis of binary traits (e.g., disease status) with binary outcomes in 2S-MR can result in inaccurate causal estimates [12]. For the primary analysis we used the Wald-ratio and random-effects inverse variance weighted method (tabulated in Supplementary Table 3) but considered weighted median, mode, and MR-Egger in sensitivity analyses, since these methods make different assumptions regarding pleiotropy and outliers [9]. Analyses were conducted using R v4.2.1. After accounting for 35 multiple comparisons using Bonferroni adjustment, a *P* < 1.42 × 10^−3^ was considered significant and a *P* < 0.05 was considered as being suggestively significant.

Our MR analyses provided no evidence to support a significant relationship between all CHIP and genetically predicted lifetime smoking, dietary intake, or circulating levels of fatty acids and lipoproteins or inflammatory factors (Supplementary Fig. 3). There was however an association shown with genetically predicted levels of vitamins B6 (odds ratio (OR) = 2.33, 95% CI: 1.20–4.53, *P* = 0.012) and B12 (OR = 1.07, 95% CI: 1.01–1.14, *P* = 0.034), as well as basal metabolic rate (OR = 1.13, 95% CI: 1.04–1.23, *P* = 3.00 × 10^−3^), albeit only at suggestive level of statistical significance.

While genetically predicted lifetime smoking [13] was not significantly associated with CHIP overall (OR = 1.12, 95% CI: 0.91–1.37, *P* = 0.304; Fig. 2), there were significant differences in the association between smoking and CHIP subtypes (Supplementary Fig. 2). Consistent with previous reports correlating smoking status with increased risk of somatic mutations [14], genetically predicted lifetime smoking was significantly associated with CHIP-*ASXL1* (OR = 4.57, 95% CI: 2.34–8.95, *P* = 9.1 × 10^-6^). Additionally, when considering mosaic chromosomal alterations, genetically predicted lifetime smoking was significantly associated with mLOY (OR = 1.70, 95% CI: 1.37–2.12, *P* = 1.7 × 10^–6^), as well as showing a suggestive association with mLOX (OR = 1.45, 95% CI: 1.08–1.94, *P* = 0.014).

**Fig. 2:**
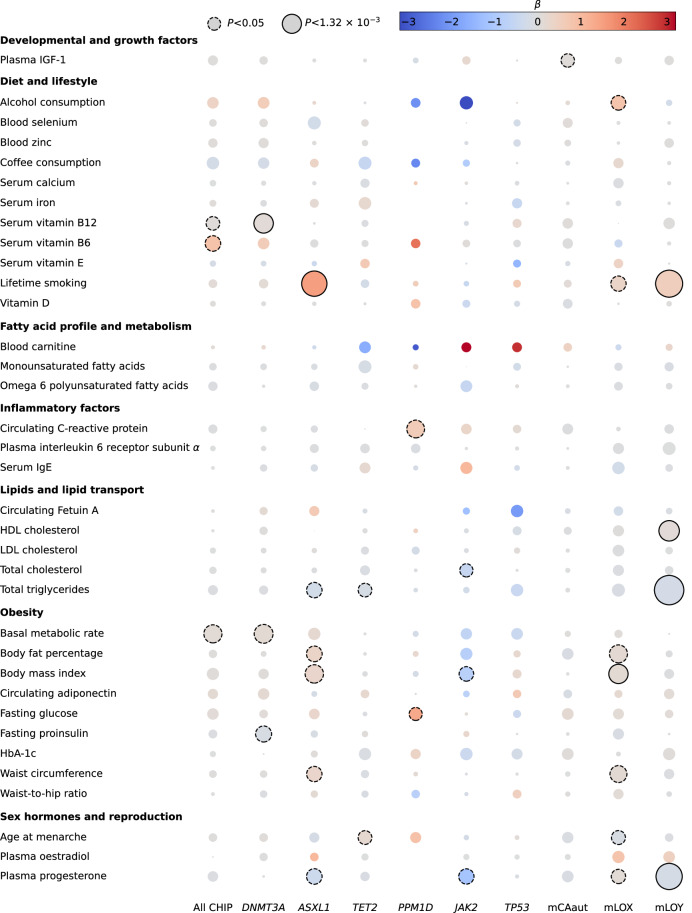
Association between modifiable potential risk factors and CHIP. The columns correspond to different CHIP subtypes and mosaic chromosomal alterations. The colours correspond to the strength of associations (log odds ratio, *β*) and their direction (red showing positive correlation, blue showing negative correlation). *P* values represent the results from two-sided tests and are unadjusted. The size of each bubble corresponds to the −log_10_ *P* value, with increasing size indicating a smaller *P* value. Dashed bubble outlines correspond to associations at *P* < 0.05 and those with solid outlines correspond to *P*_Bonferroni_ < 0.05.

Aside from the relationship with genetically predicted lifetime smoking, an association was also shown between CHIP-*DNMT3A* and genetically predicted increased levels of vitamin B12 (OR = 1.13, 95% CI: 1.05–1.21, *P* = 1.00 × 10^−3^). Since 79% of CHIP cases in our analysis were of the *DNMT3A* subtype, this relationship explains the suggestive association between vitamin B12 and all CHIP. mLOY showed a significant association with HDL-cholesterol, total triglycerides and plasma progesterone, as well as lifetime smoking (Supplementary Fig. 11). mLOX also showed a significant association with body mass index (Supplementary Fig. 10).

Inevitably the application of a Bonferroni correction can be overly stringent, because the exposures studied are likely not independent. However, while we noted suggestive associations between CHIP subtypes, mCAaut and mLOX, with obesity, lipid and lipid transport, inflammatory and hormonal factors (Fig. 2, Supplementary Figs. 4–12, and Supplementary Table 2), there was little evidence for consistent effects across subtypes.

The strength of our MR study is the exploitation of large GWAS data sets to examine the relationship between multiple phenotypes and the risk of CHIP, thereby increasing study power and enabling us to demonstrate effects of small magnitude. For 31 out of the 35 of the exposures, we had at least 80% power to demonstrate an OR per standard deviation of 1.25, stipulating a *P* value <0.05. However, we cannot exclude the possibility that the null results we observed were simply a consequence of limited study power, if the true effect of these phenotypes is marginal. A central assumption in MR is that the variants used as IVs are associated with the exposure being investigated. To ensure this was the case, we only used SNPs associated with exposure traits at genome-wide significance (*P* < 5 × 10^−8^) from GWASs. Our analysis does, however, have limitations. Firstly, we were limited to studying phenotypes with available genetic instruments. Secondly, correcting for multiple testing means the potential for false negatives is not unsubstantial. Thirdly, as many of the instrumental variables are derived from a single measurement, we are unable to study time-varying effects of exposures. Fourthly, exposures may be correlated making deconvolution of the causal risk factor problematic. Finally, not unique to our MR study, is the impact of pleiotropic IVs in our analysis. To minimise this, we incorporated information from weighted median and mode-based MR methods.

In conclusion, our study provides further insight into the landscape of CHIP aetiology. Specifically, with the exception of the known association with smoking, we find no evidence for any of the examined modifiable traits being major risk factors for the development of CHIP.

### Supplementary information

Dataset 1

Supplementary Figures

## Data Availability

Lifetime smoking GWAS summary data are publicly available for download via Wootton at al. 2020 [13]. The exposure data are available from MR base [15]. The summary statistics for clonal haematopoiesis risk are available from the GWAS Catalogue (https://www.ebi.ac.uk/gwas/publications/36450978) [3]. Analysis in this study is adapted from code available in our public repository: https://github.com/houlstonlab/MR-PheWAS. The datasets and analysis scripts are available from the corresponding author upon reasonable request.
