# Supplementary material for: Using Mendelian Randomisation to search for modifiable risk factors influencing the development of clonal haematopoiesis
Source: Blood Cancer J. 2024 Jul 16;14(1):114. doi: 10.1038/s41408-024-01101-y (PMC11252326; doi:10.1038/s41408-024-01101-y)

Supplementary Figure 1: Proportion of exposures having 80% power to demonstrate a relationship with all CHIP as a function of Odds ratio

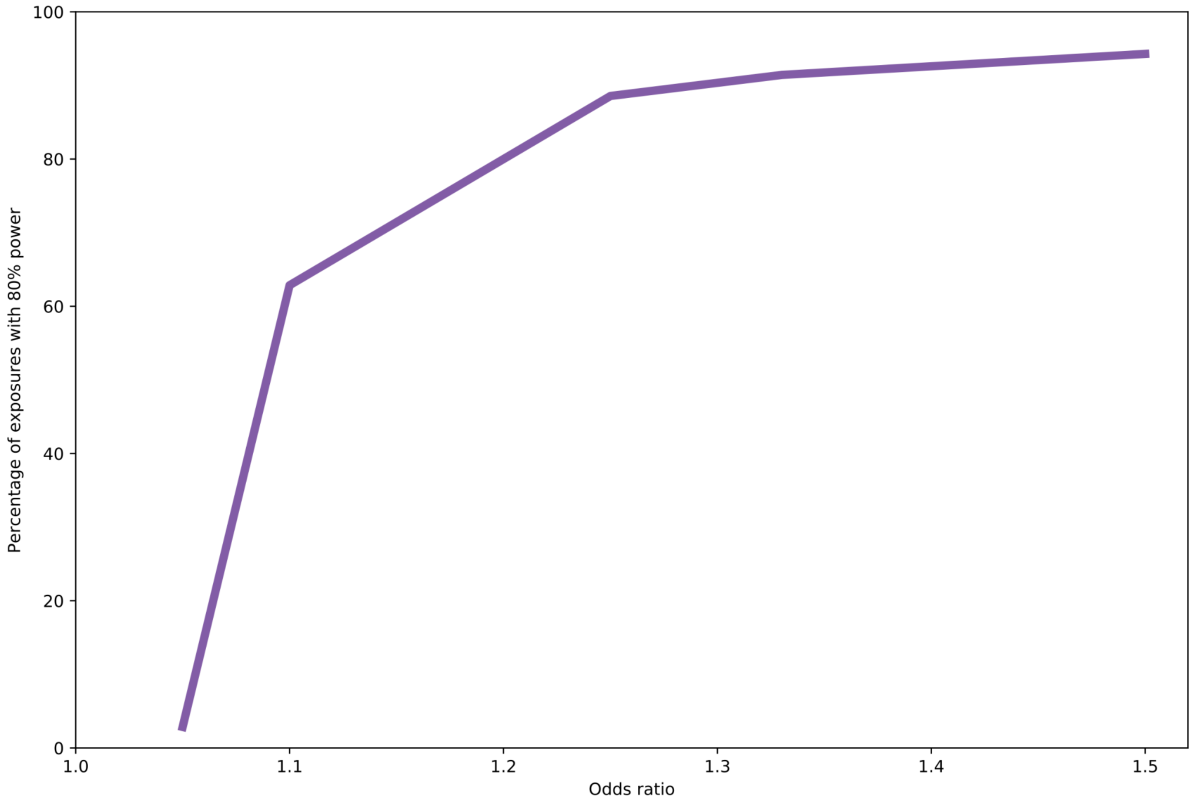

Supplementary Figure 2: Association of lifetime smoking with CHIP by subtype

Odds ratio (OR), 95% confidence intervals (CI), *P*-values and proportion of variance across SNPs due to heterogeneity (I^2) statistics for lifetime smoking for CHIP and mosaic chromosomal alterations. *ASXL1*, mLOX and mLOY show statistically significant associations with lifetime smoking. nSNP, number of single nucleotide polymorphisms.

	nSNP	*P* – value	OR (95% CI)	I^2
All CHIP	120	0.304	1.12 (0.91–1.37)	7
CHIP–DNMT3A	120	0.238	1.15 (0.91–1.46)	11
CHIP–ASXL1	120	9.1×10^{-6}	4.57 (2.34–8.95)	15
CHIP–TET2	120	0.315	0.79 (0.51–1.24)	0
CHIP–PPM1D	30	0.625	1.75 (0.18–16.7)	5
CHIP–JAK2	120	0.637	0.61 (0.08–4.68)	0
CHIP–TP53	120	0.378	1.88 (0.46–7.63)	1
mCAaut	120	0.379	1.13 (0.86–1.48)	0
mLOX	120	0.014	1.45 (1.08–1.94)	9
mLOY	120	1.7×10^{-6}	1.70 (1.37–2.12)	33

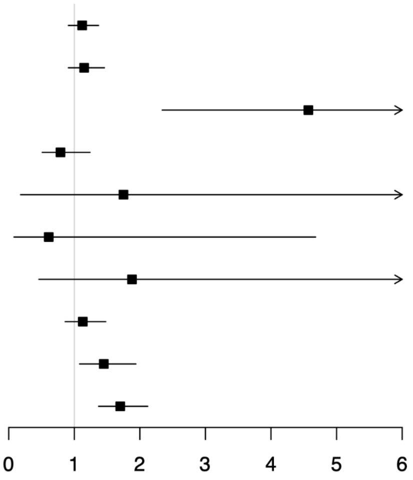

Supplementary Figure 3: Forest plot showing results for modifiable factors on All CHIP

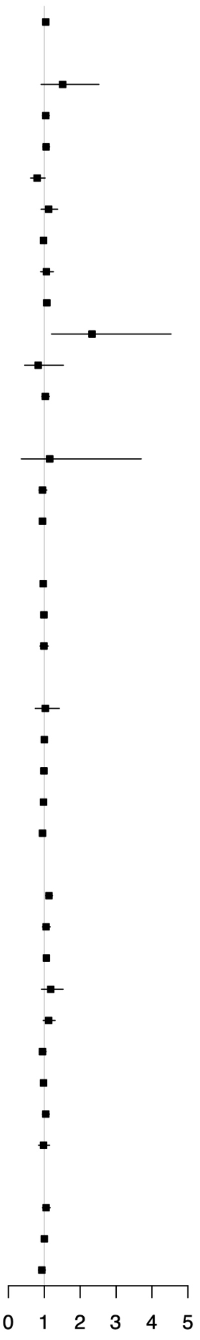

All CHIP	nSNP	*P* – value	OR (95% CI)	I^2
**Developmental and growth factors**				
Plasma IGF–1	322	0.194	1.04 (0.98–1.10)	29
**Diet and lifestyle**				
Alcohol consumption	4	0.113	1.51 (0.91–2.52)	11
Blood selenium	1	0.410	1.04 (0.95–1.14)	––
Blood zinc	2	0.244	1.05 (0.96–1.15)	36
Coffee consumption	38	0.073	0.80 (0.62–1.02)	11
Lifetime smoking	120	0.304	1.12 (0.91–1.37)	7
Serum calcium	188	0.528	0.98 (0.91–1.05)	20
Serum iron	3	0.486	1.06 (0.90–1.25)	60
Serum vitamin B12	10	0.034	1.07 (1.01–1.14)	0
Serum vitamin B6	1	0.012	2.33 (1.20–4.53)	––
Serum vitamin E	3	0.553	0.83 (0.45–1.53)	43
Vitamin D	114	0.602	1.03 (0.93–1.14)	25
**Fatty acid profile and metabolism**				
Blood carnitine	17	0.813	1.15 (0.36–3.70)	68
Monounsaturated fatty acids	7	0.414	0.95 (0.85–1.07)	46
Omega 6 polyunsaturated fatty acids	12	0.243	0.95 (0.87–1.03)	47
**Inflammatory factors**				
Circulating C–reactive protein	180	0.267	0.97 (0.92–1.02)	20
Plasma interleukin 6 receptor subunit α	2	0.626	0.99 (0.97–1.02)	0
Serum IgE	7	0.842	0.99 (0.88–1.11)	0
**Lipids and lipid transport**				
Circulating Fetuin A	1	0.842	1.03 (0.75–1.42)	––
HDL cholesterol	243	0.882	1.00 (0.95–1.06)	23
LDL cholesterol	24	0.569	0.99 (0.94–1.03)	22
Total cholesterol	23	0.435	0.98 (0.92–1.04)	34
Total triglycerides	11	0.203	0.95 (0.87–1.03)	39
**Obesity**				
Basal metabolic rate	524	0.003	1.13 (1.04–1.23)	16
Body fat percentage	379	0.330	1.05 (0.95–1.16)	19
Body mass index	490	0.085	1.06 (0.99–1.14)	20
Circulating adiponectin	10	0.182	1.18 (0.92–1.52)	37
Fasting glucose	63	0.116	1.12 (0.97–1.30)	22
Fasting proinsulin	8	0.286	0.95 (0.86–1.05)	0
HbA–1c	273	0.572	0.98 (0.93–1.04)	33
Waist circumference	360	0.394	1.04 (0.95–1.14)	22
Waist–to–hip ratio	36	0.823	0.98 (0.84–1.15)	31
**Sex hormones and reproduction**				
Age at menarche	192	0.312	1.05 (0.95–1.16)	15
Plasma oestradiol	2	0.979	1.00 (1.00–1.00)	0
Plasma progesterone	3	0.165	0.93 (0.84–1.03)	19

Supplementary Figure 4: Forest plot showing results for modifiable factors on *ASXL1*-CHIP

ASXL1	nSNP	*P* – value	OR (95% CI)	I^2
**Developmental and growth factors**				
Plasma IGF–1	318	0.808	0.98 (0.84–1.15)	8
**Diet and lifestyle**				
Alcohol consumption	4	0.823	1.30 (0.13–12.6)	56
Blood selenium	1	0.059	0.76 (0.57–1.01)	––
Blood zinc	2	0.757	1.04 (0.83–1.29)	0
Coffee consumption	38	0.295	1.48 (0.71–3.07)	0
Lifetime smoking	120	9.1×10^{-6}	4.57 (2.34–8.95)	15
Serum calcium	190	0.782	0.97 (0.80–1.19)	8
Serum iron	3	0.278	1.19 (0.87–1.65)	0
Serum vitamin B12	9	0.921	1.01 (0.83–1.23)	0
Serum vitamin B6	1	0.345	1.02 (0.98–1.07)	––
Serum vitamin E	3	0.657	0.68 (0.13–3.71)	28
Vitamin D	114	0.772	0.96 (0.72–1.28)	14
**Fatty acid profile and metabolism**				
Blood carnitine	17	0.790	0.71 (0.05–9.19)	36
Monounsaturated fatty acids	7	0.539	0.92 (0.70–1.21)	12
Omega 6 polyunsaturated fatty acids	12	0.237	0.88 (0.71–1.09)	18
**Inflammatory factors**				
Circulating C–reactive protein	179	0.449	0.94 (0.80–1.10)	19
Plasma interleukin 6 receptor subunit α	2	0.277	0.96 (0.89–1.03)	0
Serum IgE	7	0.431	0.86 (0.59–1.25)	0
**Lipids and lipid transport**				
Circulating Fetuin A	1	0.192	1.94 (0.72–5.25)	––
HDL cholesterol	243	1.000	1.00 (0.86–1.16)	13
LDL cholesterol	22	0.661	1.03 (0.89–1.20)	26
Total cholesterol	21	0.616	0.96 (0.82–1.12)	0
Total triglycerides	11	0.013	0.77 (0.62–0.95)	0
**Obesity**				
Basal metabolic rate	525	0.082	1.24 (0.97–1.59)	10
Body fat percentage	378	0.011	1.44 (1.09–1.92)	6
Body mass index	490	0.002	1.38 (1.12–1.70)	9
Circulating adiponectin	10	0.597	0.84 (0.43–1.62)	15
Fasting glucose	63	0.159	1.38 (0.88–2.16)	20
Fasting proinsulin	8	0.438	0.86 (0.59–1.25)	29
HbA–1c	272	0.469	1.05 (0.92–1.21)	1
Waist circumference	359	0.014	1.37 (1.07–1.75)	4
Waist–to–hip ratio	36	0.439	1.18 (0.78–1.78)	0
**Sex hormones and reproduction**				
Age at menarche	192	0.189	0.83 (0.63–1.10)	3
Plasma oestradiol	1	0.310	2.89 (0.37–22.4)	––
Plasma progesterone	3	0.012	0.70 (0.53–0.93)	0

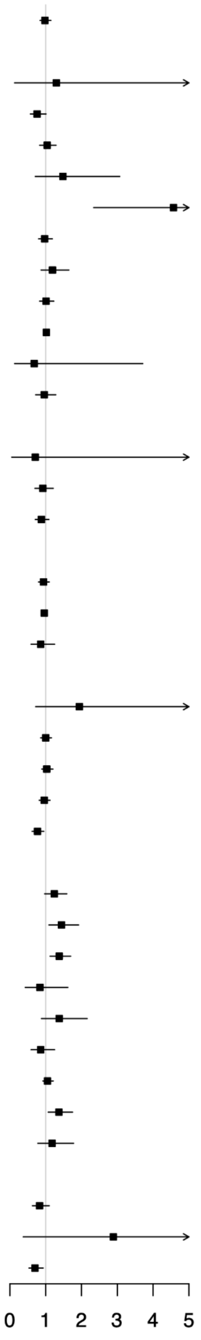

Supplementary Figure 5: Forest plot showing results for modifiable factors on *DNMT3A*-CHIP

DNMT3A	nSNP	*P* – value	OR (95% CI)	I^2
**Developmental and growth factors**				
Plasma IGF−1	318	0.329	1.03 (0.97−1.11)	34
**Diet and lifestyle**				
Alcohol consumption	4	0.103	1.81 (0.89−3.68)	42
Blood selenium	1	0.320	1.05 (0.95−1.17)	−−
Blood zinc	2	0.185	1.05 (0.97−1.14)	0
Coffee consumption	38	0.117	0.81 (0.62−1.05)	0
Lifetime smoking	120	0.238	1.15 (0.91−1.46)	11
Serum calcium	190	0.687	0.98 (0.91−1.07)	28
Serum iron	3	0.512	0.96 (0.86−1.08)	0
Serum vitamin B12	9	0.001	1.13 (1.05−1.21)	0
Serum vitamin B6	1	0.108	1.83 (0.87−3.85)	−−
Serum vitamin E	3	0.617	0.86 (0.48−1.55)	25
Vitamin D	114	0.705	0.98 (0.89−1.08)	5
**Fatty acid profile and metabolism**				
Blood carnitine	17	0.762	1.27 (0.27−6.02)	78
Monounsaturated fatty acids	7	0.505	0.97 (0.87−1.07)	16
Omega 6 polyunsaturated fatty acids	12	0.871	1.01 (0.91−1.12)	58
**Inflammatory factors**				
Circulating C−reactive protein	179	0.556	0.98 (0.93−1.04)	11
Plasma interleukin 6 receptor subunit α	2	0.652	0.99 (0.97−1.02)	0
Serum IgE	7	0.434	0.95 (0.83−1.08)	0
**Lipids and lipid transport**				
Circulating Fetuin A	1	0.351	1.19 (0.83−1.70)	−−
HDL cholesterol	242	0.336	1.03 (0.97−1.10)	34
LDL cholesterol	22	0.617	1.01 (0.96−1.07)	28
Total cholesterol	21	0.589	1.02 (0.95−1.10)	42
Total triglycerides	11	0.264	0.95 (0.87−1.04)	31
**Obesity**				
Basal metabolic rate	524	0.002	1.17 (1.06−1.29)	27
Body fat percentage	378	0.604	1.03 (0.92−1.16)	27
Body mass index	490	0.174	1.06 (0.97−1.15)	26
Circulating adiponectin	10	0.117	1.22 (0.95−1.56)	21
Fasting glucose	63	0.309	1.08 (0.93−1.26)	11
Fasting proinsulin	8	0.009	0.86 (0.77−0.96)	0
HbA−1c	271	0.977	1.00 (0.94−1.07)	38
Waist circumference	359	0.423	1.04 (0.94−1.16)	30
Waist−to−hip ratio	36	0.422	0.94 (0.79−1.10)	17
**Sex hormones and reproduction**				
Age at menarche	192	0.299	1.06 (0.95−1.20)	31
Plasma oestradiol	1	0.339	1.00 (1.00−1.00)	−−
Plasma progesterone	3	0.922	1.01 (0.91−1.11)	0

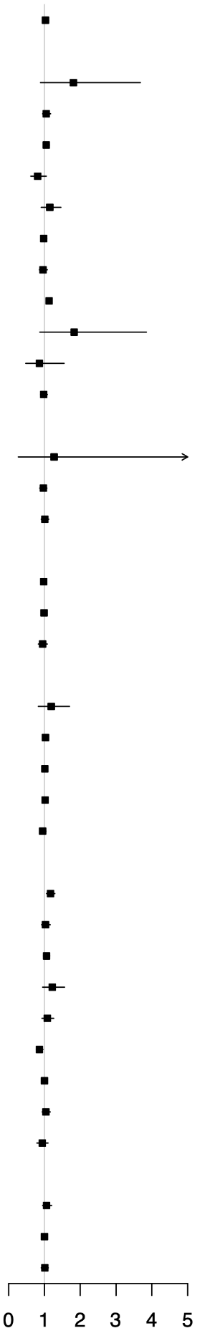

Supplementary Figure 6: Forest plot showing results for modifiable factors on *TET2*-CHIP

TET2	nSNP	*P* – value	OR (95% CI)	I^2
**Developmental and growth factors**				
Plasma IGF−1	318	0.815	0.99 (0.88−1.11)	10
**Diet and lifestyle**				
Alcohol consumption	4	0.912	0.94 (0.32−2.77)	0
Blood selenium	1	0.416	1.09 (0.89−1.33)	−−
Blood zinc	2	0.544	1.05 (0.90−1.23)	0
Coffee consumption	38	0.061	0.61 (0.36−1.02)	0
Lifetime smoking	120	0.315	0.79 (0.51−1.24)	0
Serum calcium	190	0.257	0.92 (0.80−1.06)	0
Serum iron	3	0.075	1.28 (0.97−1.68)	31
Serum vitamin B12	9	0.468	0.95 (0.82−1.09)	0
Serum vitamin B6	1	0.466	1.01 (0.98−1.04)	−−
Serum vitamin E	3	0.237	1.99 (0.64−6.19)	18
Vitamin D	114	0.891	1.02 (0.81−1.27)	22
**Fatty acid profile and metabolism**				
Blood carnitine	17	0.102	0.20 (0.03−1.38)	44
Monounsaturated fatty acids	7	0.064	0.84 (0.70−1.01)	0
Omega 6 polyunsaturated fatty acids	12	0.467	0.95 (0.82−1.10)	12
**Inflammatory factors**				
Circulating C−reactive protein	179	0.987	1.00 (0.90−1.12)	6
Plasma interleukin 6 receptor subunit α	2	0.226	0.97 (0.92−1.02)	0
Serum IgE	7	0.143	1.22 (0.93−1.60)	0
**Lipids and lipid transport**				
Circulating Fetuin A	1	0.708	0.87 (0.43−1.78)	−−
HDL cholesterol	242	0.677	1.02 (0.93−1.13)	0
LDL cholesterol	22	0.357	0.95 (0.85−1.06)	28
Total cholesterol	21	0.202	0.91 (0.80−1.05)	34
Total triglycerides	11	0.033	0.85 (0.73−0.99)	0
**Obesity**				
Basal metabolic rate	525	0.896	1.01 (0.85−1.20)	2
Body fat percentage	378	0.847	1.02 (0.84−1.25)	2
Body mass index	490	0.647	0.97 (0.84−1.12)	5
Circulating adiponectin	10	0.306	1.26 (0.81−1.95)	0
Fasting glucose	63	0.729	0.95 (0.71−1.27)	0
Fasting proinsulin	8	0.542	1.07 (0.86−1.34)	0
HbA−1c	272	0.083	0.91 (0.83−1.01)	7
Waist circumference	359	0.341	0.92 (0.77−1.09)	0
Waist−to−hip ratio	36	0.449	0.89 (0.66−1.20)	0
**Sex hormones and reproduction**				
Age at menarche	192	0.032	1.25 (1.02−1.52)	5
Plasma oestradiol	1	0.456	1.00 (1.00−1.00)	−−
Plasma progesterone	3	0.241	0.89 (0.72−1.08)	0

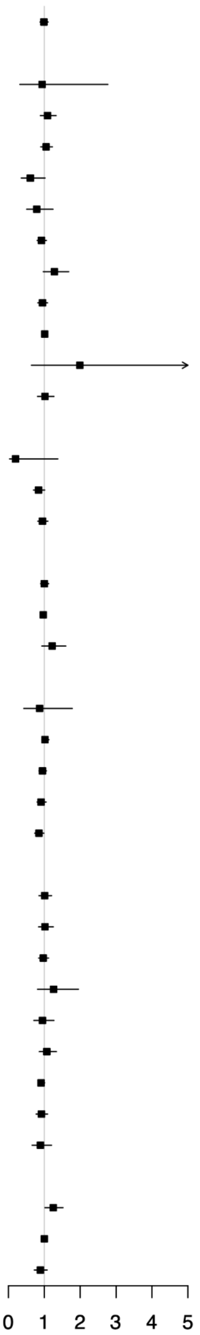

Supplementary Figure 7: Forest plot showing results for modifiable factors on *JAK2*-CHIP

JAK2	nSNP	*P* – value	OR (95% CI)	I^2
**Developmental and growth factors**				
Plasma IGF−1	318	0.330	1.28 (0.78−2.09)	1
**Diet and lifestyle**				
Alcohol consumption	4	0.054	0.00 (0.00−1.12)	50
Blood selenium	1	0.982	1.01 (0.40−2.54)	−−
Blood zinc	2	0.794	0.89 (0.37−2.13)	33
Coffee consumption	38	0.450	0.40 (0.04−4.33)	0
Lifetime smoking	120	0.637	0.61 (0.08−4.68)	0
Serum calcium	190	0.855	1.06 (0.55−2.07)	14
Serum iron	3	0.918	0.95 (0.32−2.75)	0
Serum vitamin B12	9	0.695	0.88 (0.45−1.69)	1
Serum vitamin B6	1	0.398	1.06 (0.92−1.23)	−−
Serum vitamin E	3	0.960	0.81 (0.00−999)	68
Vitamin D	114	0.373	0.67 (0.28−1.62)	0
**Fatty acid profile and metabolism**				
Blood carnitine	17	0.205	72.6 (0.10−999)	0
Monounsaturated fatty acids	7	0.999	1.00 (0.35−2.85)	36
Omega 6 polyunsaturated fatty acids	12	0.100	0.59 (0.32−1.11)	0
**Inflammatory factors**				
Circulating C−reactive protein	179	0.161	1.41 (0.87−2.27)	0
Plasma interleukin 6 receptor subunit α	2	0.874	0.98 (0.72−1.33)	34
Serum IgE	7	0.091	2.89 (0.84−9.90)	0
**Lipids and lipid transport**				
Circulating Fetuin A	1	0.455	0.29 (0.01−7.46)	−−
HDL cholesterol	243	0.708	0.91 (0.57−1.46)	8
LDL cholesterol	22	0.707	0.92 (0.60−1.41)	2
Total cholesterol	21	0.042	0.60 (0.36−0.98)	0
Total triglycerides	11	0.532	0.78 (0.35−1.71)	26
**Obesity**				
Basal metabolic rate	525	0.122	0.55 (0.26−1.17)	0
Body fat percentage	378	0.088	0.45 (0.18−1.13)	5
Body mass index	490	0.021	0.46 (0.24−0.89)	5
Circulating adiponectin	10	0.508	0.48 (0.06−4.19)	14
Fasting glucose	63	0.867	1.14 (0.24−5.39)	29
Fasting proinsulin	8	0.570	1.35 (0.48−3.75)	0
HbA−1c	272	0.079	0.67 (0.42−1.05)	2
Waist circumference	359	0.899	0.95 (0.43−2.11)	1
Waist−to−hip ratio	36	0.793	0.83 (0.21−3.32)	5
**Sex hormones and reproduction**				
Age at menarche	192	0.650	0.81 (0.33−1.98)	0
Plasma oestradiol	1	0.570	1.00 (0.98−1.01)	−−
Plasma progesterone	3	0.014	0.32 (0.13−0.80)	0

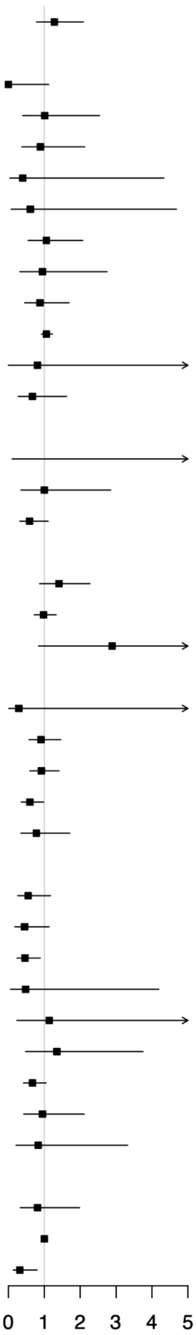

Supplementary Figure 8: Forest plot showing results for modifiable factors on *PPM1D*-CHIP

PPM1D	nSNP	*P* – value	OR (95% CI)	I^2
**Developmental and growth factors**				
Plasma IGF−1	68	0.605	0.84 (0.44−1.62)	16
**Diet and lifestyle**				
Alcohol consumption	2	0.222	0.12 (0.00−3.67)	0
Coffee consumption	6	0.283	0.10 (0.00−6.61)	0
Lifetime smoking	30	0.625	1.75 (0.18−16.7)	5
Serum calcium	49	0.816	2.01 (0.01−725)	0
Serum vitamin B6	1	0.235	8.78 (0.24−316)	−−
Vitamin D	28	0.233	2.30 (0.59−9.05)	17
**Fatty acid profile and metabolism**				
Blood carnitine	3	0.584	0.05 (0.00−999)	0
Monounsaturated fatty acids	2	0.641	1.23 (0.52−2.90)	17
Omega 6 polyunsaturated fatty acids	4	0.871	1.06 (0.51−2.21)	0
**Inflammatory factors**				
Circulating C−reactive protein	49	0.003	1.85 (1.23−2.78)	0
Plasma interleukin 6 receptor subunit α	2	0.231	0.92 (0.81−1.05)	0
Serum IgE	3	0.685	0.83 (0.35−2.01)	0
**Lipids and lipid transport**				
HDL cholesterol	46	0.730	1.54 (0.13−18.2)	37
LDL cholesterol	9	0.366	0.78 (0.46−1.33)	38
Total cholesterol	9	0.831	0.94 (0.51−1.72)	38
Total triglycerides	3	0.743	0.84 (0.30−2.34)	54
**Obesity**				
Basal metabolic rate	116	0.581	0.78 (0.33−1.87)	0
Body fat percentage	96	0.597	1.31 (0.48−3.56)	12
Body mass index	110	0.586	0.82 (0.40−1.67)	1
Circulating adiponectin	2	0.950	0.91 (0.04−20.2)	0
Fasting glucose	13	0.046	3.84 (1.03−14.3)	0
HbA−1c	55	0.185	1.47 (0.83−2.62)	5
Waist circumference	90	0.734	1.16 (0.49−2.78)	6
Waist−to−hip ratio	12	0.318	0.46 (0.10−2.10)	30
**Sex hormones and reproduction**				
Age at menarche	43	0.135	2.37 (0.76−7.38)	25

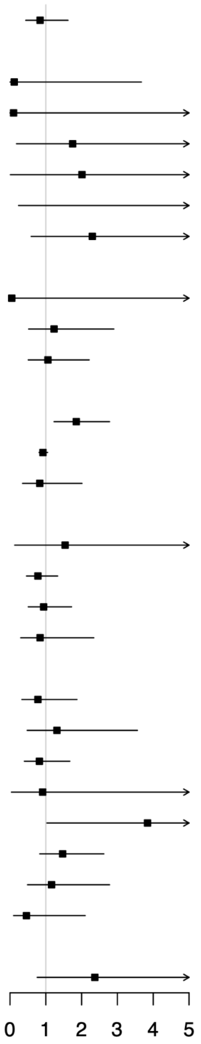

Supplementary Figure 9: Forest plot showing results for modifiable factors on *TP53*-CHIP

TP53	nSNP	*P* – value	OR (95% CI)	I^2
**Developmental and growth factors**				
Plasma IGF−1	318	0.952	0.99 (0.71−1.39)	0
**Diet and lifestyle**				
Alcohol consumption	4	0.960	1.14 (0.01−194)	57
Blood selenium	1	0.458	0.79 (0.42−1.48)	−−
Blood zinc	2	0.421	0.82 (0.50−1.34)	0
Coffee consumption	38	0.933	0.93 (0.17−5.14)	9
Lifetime smoking	120	0.378	1.88 (0.46−7.63)	1
Serum calcium	190	0.740	0.93 (0.60−1.43)	0
Serum iron	3	0.187	0.61 (0.30−1.27)	0
Serum vitamin B12	9	0.274	1.28 (0.82−2.01)	0
Serum vitamin B6	1	0.406	0.96 (0.87−1.06)	−−
Serum vitamin E	3	0.374	0.23 (0.01−5.78)	0
Vitamin D	114	0.568	0.84 (0.45−1.54)	0
**Fatty acid profile and metabolism**				
Blood carnitine	17	0.211	18.3 (0.19−999)	0
Monounsaturated fatty acids	7	0.387	0.77 (0.42−1.40)	9
Omega 6 polyunsaturated fatty acids	12	0.812	1.05 (0.68−1.62)	0
**Inflammatory factors**				
Circulating C−reactive protein	179	0.322	1.18 (0.85−1.64)	0
Plasma interleukin 6 receptor subunit α	2	0.795	0.98 (0.82−1.16)	0
Serum IgE	7	0.620	0.77 (0.27−2.19)	35
**Lipids and lipid transport**				
Circulating Fetuin A	1	0.079	0.14 (0.01−1.26)	−−
HDL cholesterol	243	0.281	0.84 (0.62−1.15)	0
LDL cholesterol	22	0.556	1.09 (0.82−1.46)	0
Total cholesterol	21	0.902	1.02 (0.72−1.44)	0
Total triglycerides	11	0.078	0.64 (0.39−1.05)	15
**Obesity**				
Basal metabolic rate	525	0.105	0.65 (0.38−1.09)	0
Body fat percentage	378	0.448	1.27 (0.69−2.35)	0
Body mass index	490	0.275	1.28 (0.82−1.99)	0
Circulating adiponectin	10	0.327	1.98 (0.51−7.75)	0
Fasting glucose	63	0.365	0.66 (0.27−1.62)	0
Fasting proinsulin	8	0.940	1.03 (0.51−2.07)	0
HbA−1c	272	0.138	0.79 (0.58−1.08)	1
Waist circumference	359	0.879	0.96 (0.56−1.65)	0
Waist−to−hip ratio	36	0.285	1.66 (0.66−4.19)	0
**Sex hormones and reproduction**				
Age at menarche	192	0.732	1.11 (0.60−2.06)	1
Plasma oestradiol	1	0.675	1.00 (0.99−1.01)	−−
Plasma progesterone	3	0.796	0.92 (0.49−1.73)	0

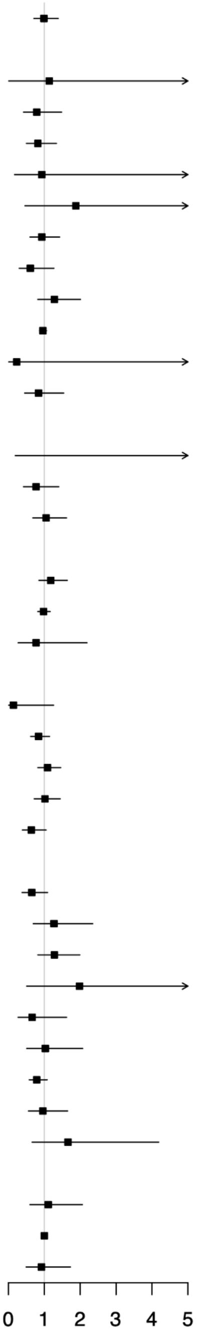

Supplementary Figure 10: Forest plot showing results for modifiable factors on mosaic loss-of-X chromosome

Mosaic loss–of–X chromosome	nSNP	*P* – value	OR (95% CI)	I^2
**Developmental and growth factors**				
Plasma IGF−1	317	0.446	1.03 (0.96–1.11)	16
**Diet and lifestyle**				
Alcohol consumption	4	0.021	2.23 (1.13–4.39)	0
Blood selenium	1	0.819	0.99 (0.87–1.12)	––
Blood zinc	2	0.856	0.98 (0.80–1.21)	77
Coffee consumption	38	0.181	1.26 (0.90–1.77)	6
Lifetime smoking	120	0.014	1.45 (1.08–1.94)	9
Serum calcium	190	0.175	0.94 (0.86–1.03)	9
Serum iron	3	0.615	0.92 (0.68–1.26)	77
Serum vitamin B12	9	0.995	1.00 (0.90–1.11)	20
Serum vitamin B6	1	0.357	0.65 (0.26–1.64)	––
Serum vitamin E	3	0.237	1.49 (0.77–2.90)	5
Vitamin D	114	0.932	0.99 (0.86–1.15)	25
**Fatty acid profile and metabolism**				
Blood carnitine	17	0.578	0.70 (0.20–2.44)	45
Monounsaturated fatty acids	7	0.341	0.91 (0.76–1.10)	62
Omega 6 polyunsaturated fatty acids	12	0.299	0.94 (0.83–1.06)	46
**Inflammatory factors**				
Circulating C−reactive protein	179	0.747	1.01 (0.94–1.10)	29
Plasma interleukin 6 receptor subunit α	2	0.132	0.97 (0.94–1.01)	0
Serum IgE	7	0.078	0.77 (0.57–1.03)	66
**Lipids and lipid transport**				
Circulating Fetuin A	1	0.234	0.76 (0.48–1.19)	––
HDL cholesterol	243	0.131	1.05 (0.99–1.12)	7
LDL cholesterol	22	0.108	0.95 (0.90–1.01)	0
Total cholesterol	21	0.134	0.94 (0.87–1.02)	29
Total triglycerides	11	0.060	0.88 (0.78–1.01)	47
**Obesity**				
Basal metabolic rate	525	0.349	1.06 (0.94–1.19)	20
Body fat percentage	378	0.003	1.21 (1.07–1.38)	3
Body mass index	490	0.001	1.16 (1.06–1.27)	4
Circulating adiponectin	10	0.361	1.23 (0.79–1.90)	60
Fasting glucose	63	0.142	1.14 (0.96–1.37)	0
Fasting proinsulin	8	0.132	0.89 (0.76–1.04)	20
HbA−1c	272	0.721	1.01 (0.94–1.09)	27
Waist circumference	359	0.005	1.17 (1.05–1.30)	0
Waist−to−hip ratio	36	0.183	1.15 (0.94–1.40)	13
**Sex hormones and reproduction**				
Age at menarche	192	0.028	0.86 (0.76–0.98)	13
Plasma oestradiol	1	0.085	2.25 (0.89–5.64)	––
Plasma progesterone	3	0.024	1.16 (1.02–1.31)	0

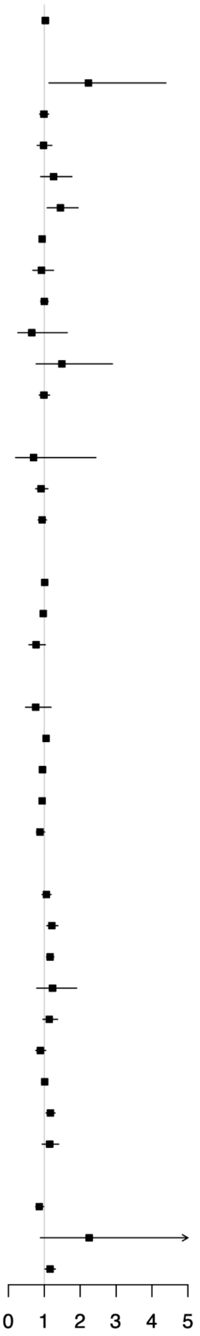

Supplementary Figure 11: Forest plot showing results for modifiable factors on mosaic loss-of-Y chromosome

Mosaic loss–of–Y chromosome	nSNP	*P* – value	OR (95% CI)	I^2
**Developmental and growth factors**				
Plasma IGF–1	316	0.240	1.04 (0.97–1.11)	55
**Diet and lifestyle**				
Alcohol consumption	4	0.549	0.77 (0.33–1.80)	74
Blood selenium	1	0.842	0.99 (0.91–1.08)	––
Blood zinc	2	0.229	1.04 (0.98–1.11)	0
Coffee consumption	38	0.794	1.05 (0.74–1.49)	64
Lifetime smoking	120	1.7×10^{-6}	1.70 (1.37–2.12)	33
Serum calcium	190	0.797	1.01 (0.92–1.11)	63
Serum iron	3	0.861	1.02 (0.83–1.24)	79
Serum vitamin B12	9	0.114	1.05 (0.99–1.11)	0
Serum vitamin B6	1	0.793	1.00 (0.99–1.01)	––
Serum vitamin E	3	0.946	1.05 (0.27–4.03)	91
Vitamin D	114	0.642	1.03 (0.91–1.16)	59
**Fatty acid profile and metabolism**				
Blood carnitine	17	0.466	1.35 (0.60–3.06)	49
Monounsaturated fatty acids	7	0.259	0.90 (0.75–1.08)	84
Omega 6 polyunsaturated fatty acids	12	0.523	0.97 (0.88–1.06)	65
**Inflammatory factors**				
Circulating C–reactive protein	179	0.235	0.96 (0.91–1.02)	51
Plasma interleukin 6 receptor subunit α	2	0.062	0.98 (0.96–1.00)	0
Serum IgE	7	0.407	0.94 (0.81–1.09)	44
**Lipids and lipid transport**				
Circulating Fetuin A	1	0.517	0.91 (0.69–1.21)	––
HDL cholesterol	243	0.001	1.12 (1.05–1.19)	61
LDL cholesterol	23	0.416	0.98 (0.93–1.03)	55
Total cholesterol	22	0.314	0.96 (0.89–1.04)	65
Total triglycerides	11	1.5×10^{-7}	0.81 (0.75–0.88)	43
**Obesity**				
Basal metabolic rate	522	0.968	1.00 (0.91–1.11)	53
Body fat percentage	377	0.471	0.96 (0.86–1.07)	49
Body mass index	488	0.209	0.95 (0.88–1.03)	46
Circulating adiponectin	10	0.176	1.14 (0.94–1.39)	19
Fasting glucose	63	0.327	1.11 (0.90–1.37)	71
Fasting proinsulin	8	0.925	0.99 (0.88–1.12)	42
HbA–1c	272	0.083	1.06 (0.99–1.14)	67
Waist circumference	358	0.182	0.94 (0.86–1.03)	41
Waist–to–hip ratio	36	0.633	0.96 (0.80–1.15)	57
**Sex hormones and reproduction**				
Age at menarche	192	0.318	0.94 (0.84–1.06)	53
Plasma oestradiol	1	0.106	1.62 (0.90–2.91)	––
Plasma progesterone	3	3.9×10^{-6}	0.83 (0.77–0.90)	0

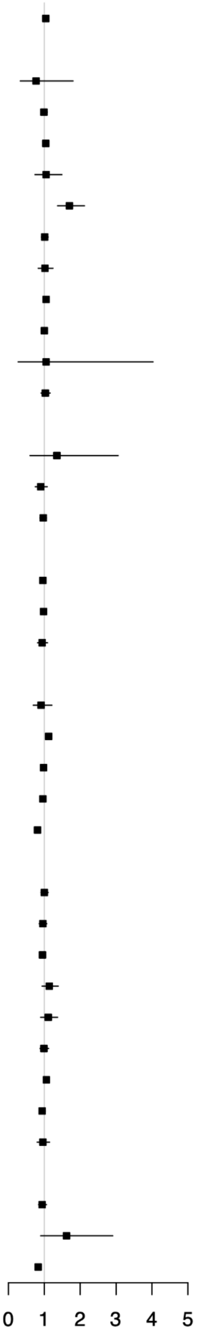

Supplementary Figure 12: Forest plot showing results for modifiable factors on autosomal mosaic chromosomal alterations

Autosomal mosaic chromosomal alterations	nSNP	$P-$value	OR (95% CI)	I^2
**Developmental and growth factors**				
Plasma IGF−1	318	0.038	1.07 (1.00−1.15)	0
**Diet and lifestyle**				
Alcohol consumption	4	0.744	1.18 (0.44−3.14)	56
Blood selenium	1	0.198	1.08 (0.96−1.23)	−−
Blood zinc	2	0.503	1.03 (0.94−1.14)	0
Coffee consumption	38	0.692	0.94 (0.68−1.29)	0
Lifetime smoking	120	0.379	1.13 (0.86−1.48)	0
Serum calcium	190	0.914	1.00 (0.91−1.08)	3
Serum iron	3	0.829	0.98 (0.85−1.13)	0
Serum vitamin B12	9	0.165	1.06 (0.97−1.16)	0
Serum vitamin B6	1	0.169	1.01 (0.99−1.03)	−−
Serum vitamin E	3	0.672	0.87 (0.47−1.63)	0
Vitamin D	114	0.214	0.93 (0.82−1.04)	0
**Fatty acid profile and metabolism**				
Blood carnitine	17	0.312	1.58 (0.65−3.87)	0
Monounsaturated fatty acids	7	0.935	1.00 (0.89−1.11)	0
Omega 6 polyunsaturated fatty acids	12	0.824	0.99 (0.88−1.10)	44
**Inflammatory factors**				
Circulating C−reactive protein	179	0.146	0.95 (0.89−1.02)	1
Plasma interleukin 6 receptor subunit α	2	0.911	1.00 (0.97−1.03)	0
Serum IgE	7	0.826	1.02 (0.86−1.20)	0
**Lipids and lipid transport**				
Circulating Fetuin A	1	0.617	0.89 (0.58−1.38)	−−
HDL cholesterol	243	0.376	0.97 (0.92−1.03)	3
LDL cholesterol	22	0.782	0.99 (0.93−1.06)	32
Total cholesterol	21	0.690	0.98 (0.90−1.07)	39
Total triglycerides	11	0.726	1.02 (0.92−1.13)	19
**Obesity**				
Basal metabolic rate	525	0.561	1.03 (0.93−1.15)	14
Body fat percentage	378	0.112	0.90 (0.80−1.02)	9
Body mass index	490	0.860	0.99 (0.91−1.08)	2
Circulating adiponectin	10	0.295	0.86 (0.65−1.14)	7
Fasting glucose	63	0.103	1.19 (0.96−1.48)	32
Fasting proinsulin	8	0.840	0.98 (0.83−1.17)	36
HbA−1c	272	0.102	1.05 (0.99−1.12)	5
Waist circumference	359	0.653	0.98 (0.88−1.09)	1
Waist−to−hip ratio	36	0.670	1.04 (0.87−1.25)	0
**Sex hormones and reproduction**				
Age at menarche	192	0.121	0.91 (0.81−1.03)	0
Plasma oestradiol	1	0.594	1.00 (1.00−1.00)	−−
Plasma progesterone	3	0.106	0.89 (0.77−1.03)	27

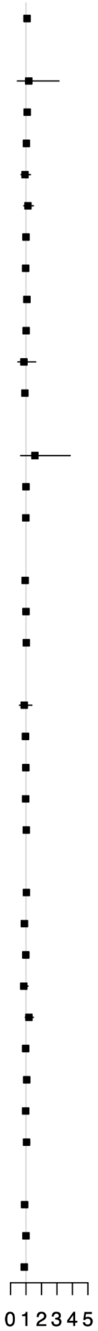

Supplement: Supplementary file 2 — Supplementary Figures [file 41408_2024_1101_MOESM2_ESM.pdf]
